# Assessing Message Deployment During Public Health Emergencies Through Social Media: Empirical Test of Optimizing Content for Effective Dissemination

**DOI:** 10.2196/50871

**Published:** 2024-07-26

**Authors:** Paola Pascual-Ferrá, Neil Alperstein, Julia Burleson, Amelia M Jamison, Ananya Bhaktaram, Sidharth Rath, Rohini Ganjoo, Satyanarayan Mohanty, Daniel J Barnett, Rajiv N Rimal

**Affiliations:** 1 Loyola University Maryland Baltimore, MD United States; 2 Health, Behavior and Society, Johns Hopkins Bloomberg School of Public Health Baltimore, MD United States; 3 Swasthya Plus Network Bhubaneswar, OD India; 4 Biomedical Laboratory Sciences George Washington University Washington, DC United States; 5 D-COR Consulting Pvt Ltd Bhubaneswar, OD India; 6 Environmental Health and Engineering, Johns Hopkins Bloomberg School of Public Health Baltimore, MD United States

**Keywords:** message testing, web-based communication, user engagement, vaccine communication, methodology, Meta, Facebook, advertising, infodemic, communication, infodemiology, social media advertising tool, social media, audience, engagement, rapid message testing at scale, mobile phone

## Abstract

**Background:**

During an infodemic, timely, reliable, and accessible information is crucial to combat the proliferation of health misinformation. While message testing can provide vital information to make data-informed decisions, traditional methods tend to be time- and resource-intensive. Recognizing this need, we developed the rapid message testing at scale (RMTS) approach to allow communicators to repurpose existing social media advertising tools and understand the full spectrum of audience engagement.

**Objective:**

We had two main objectives: (1) to demonstrate the use of the RMTS approach for message testing, especially when resources and time are limited, and (2) to propose and test the efficacy of an outcome variable that measures engagement along a continuum of viewing experience.

**Methods:**

We developed 12 versions of a single video created for a vaccine confidence project in India. We manipulated video length, aspect ratio, and use of subtitles. The videos were tested across 4 demographic groups (women or men, younger or older). We assessed user engagement along a continuum of viewing experience: obtaining attention, sustaining attention, conveying the message, and inspiring action. These were measured by the percentage of video watched and clicks on the call-to-action link.

**Results:**

The video advertisements were placed on Facebook for over 4 consecutive days at the cost of US $450 and garnered a total of 3.34 million impressions. Overall, we found that the best-performing video was the shorter version in portrait aspect ratio and without subtitles. There was a significant but small association between the length of the video and users’ level of engagement at key points along the continuum of viewing experience (N=1,032,888; *χ*^2^_4_=48,261.97; *P*<.001; *V*=.22). We found that for the longer video, those with subtitles held viewers longer after 25% video watch time than those without subtitles (n=15,597; *χ*^2^_1_=7.33; *P*=.007; *V*=.02). While we found some significant associations between the aspect ratio, the use of subtitles, and the number of users watching the video and clicking on the call-to-action link, the effect size for those were extremely small.

**Conclusions:**

This test served as a proof of concept for the RMTS approach. We obtained rapid feedback on formal message attributes from a very large sample. The results of this test reinforce the need for platform-specific tailoring of communications. While our data showed a general preference for a short video in portrait orientation and without subtitles among our target audiences on Facebook, that may not necessarily be the case in other social media platforms such as YouTube or TikTok, where users go primarily to watch videos. RMTS testing highlights nuances that communication professionals can address instead of being limited to a “one size fits all” approach.

## Introduction

### Background

Message testing broadly refers to the practice of using data to make informed decisions about what is the most effective way of conveying a message to a specific target audience in a particular geographical area and cultural context to elicit a desired response. The World Health Organization recommends that health communicators engage in message testing as an important step to increase the efficacy of health campaigns, particularly during public health emergencies [1].

During the COVID-19 pandemic and accompanying infodemic, a deluge of false, misleading, and unreliable health claims obscured credible public health messaging, creating a “simultaneous crises of epidemics and misinformation” [2]. The term infodemic is defined as “an information epidemic that can lead to engaging in dangerous behavior” during a public health emergency [3]. For example, Hu et al [4] found that during the COVID-19 infodemic, there was a “global profusion of monikers and hashtags for COVID-19...[that] contributed to a backlash against China and the Chinese people.” The increased speed and reach with which misinformation spread via social media platforms exacerbated the infodemic. To that end, Hang et al [5] concluded the pandemic brought on an “unprecedented infodemic of false or misleading information that spread rapidly through online social networks.” In a joint statement, the World Health Organization along with the United Nations, UNICEF (United Nations Children’s Fund), and other international organizations emphasized that it is essential for public health messaging during an infodemic to be timely, accurate, and resonant to counter misinformation and disinformation [6].

Message testing can be a vital tool to help communicators counter misinformation, particularly as evidence emerges to support the relationship between quality health communication and increased public trust [7,8]. Message testing can be accomplished using both traditional quantitative (eg, controlled message tests) and qualitative (eg, focus groups) methods. However, acknowledging that falsehoods can often go viral, traditional message testing methods may not generate evidence fast enough to compete in an increasingly crowded and rapidly evolving information ecosystem [9]. Given the resource-intensive nature of traditional message testing and the urgency to respond quickly during public health emergencies, our team recognized the need for more accessible methods for public health practitioners. Rapid message testing at scale (RMTS) is an approach that can help public health professionals and communicators optimize content through rapid testing that is both inexpensive and uses large samples of data to promote evidence-based decision-making.

The RMTS approach works by using data from social media platforms and repurposing them to make evidence-based communication decisions. Furthermore, it recognizes that viewer attention is not binary but falls along a spectrum. We developed a measure of the continuum of viewing experience measures to assess audience engagement and test the RMTS approach. This allowed us to assess differences in user engagement related to 3 content-agnostic metrics: aspect ratio, video length, and subtitle use across different audience types. In addition to testing the RMTS approach, the results of this analysis produced valuable information for users looking to improve advertisement performance on social media more generally, particularly when adapting content from other media formats such as television advertisements, thus helping organizations increase their return on investment.

### Web-Based Message Testing

Due to the low barrier to entry and potential for immediate and widespread dissemination, social media is often viewed as a cost-effective vehicle for a communication campaign, especially when time and reach are of the essence, such as during an infodemic. Platforms such as Google and Meta have created user-friendly interfaces that make low-cost digital advertising accessible to almost anyone with a credit card. Many of these services offer data for users to make sense of audience engagement and select specific advertisement features. While placing video advertisements on social media can be less expensive than paying for television advertisements, the number of available platforms—each with its own features and formats—makes designing messages quite complex. For example, on some platforms, a horizontal aspect ratio for viewing videos is preferred (YouTube, Google LLC), while on others vertical is preferred (Instagram, Meta Platforms, Inc, or Facebook, Meta Platforms, Inc). This may seem trivial at first glance, but it is important to recognize that there is agency in viewing videos on digital media, making it easy to move on to the next video. In other words, the format may impact whether the video is watched at all, viewed for a few seconds or longer, or results in repeat viewing. Video length is another factor to be considered. Traditional public service announcements on television may last up to 2 minutes. However, in the digital age, many people watch only a few seconds of a video before moving on to other content; thus, structuring a video to grab and keep the attention of the viewer is paramount. This is not to negate the importance of compelling and theoretically developed content. Rather, we propose that, in a digital environment, format attributes and formal features can make a difference in terms of sustaining attention and user engagement.

With video content in particular, the issue of viewer abandonment—abandoning a video before watching it in its entirety—is one of the greatest challenges for communicators. Researchers who studied watch time for short videos (5 minutes or less) on YouTube found that on average, 20% of viewers abandoned the video within the first 10 seconds, 33% abandoned the video within the first 30 seconds, and 44% of viewers did so within the first 60 seconds [10]. This is an important consideration when designing and creating video content for social media.

Another challenge when placing advertisements on social media platforms is banner blindness, a form of practiced avoidance in which individuals turn away from advertising content that appears on the screen [11]. It is all too easy to equate exposure to social media with awareness. However, distracted viewing or watching a message with split attention—when users’ eyes are on the screen but their minds are elsewhere—has become commonplace in the digital world. For that matter, the amount of views and likes a video receives are considered superficial or vanity metrics that may not accurately reflect the quality of user engagement [12]. The issue of attention span extends beyond the user to consider the platform itself and how its algorithms’ video length preferences affect what content is suggested to viewers [13]. The viewing experience on digital platforms is complex as viewing situations are different for different media. For instance, while sound, sound effects, and music may be important on a specific medium or in a private (home) viewing situation, in other more public viewing situations, a video whose message comes through without sound may be the preferred option [14,15]. Multimedia Appendix 1 provides a glossary of terms relevant to our study.

### Assessing User Engagement

With the RMTS approach, we sought to move beyond vanity metrics to develop a model of more nuanced metrics based on a continuum of viewing experience. The approach is based on A/B testing premises, a standard in marketing practice that works well within the bounds of digital media. The practice is commonly used to test which 1 of 2 options (in our case, video advertisements) performs better among specific target audiences to make an evidence-based decision before investing significant advertising resources. Public health communicators are beginning to adopt this marketing industry approach by using A/B testing [16]. For instance, Facebook teamed up with UNICEF during the COVID-19 pandemic to test the effectiveness of different messages in building vaccine confidence using their social media platform [17]. Their research formed the basis for the Vaccine Demand Observatory, a collaboration between UNICEF, The Public Goods Projects, and Yale University’s Institute for Global Health to help counter vaccine misinformation using social listening analytics [18]. Their research confirmed the ability to effectively microtarget vaccine promotion messaging to specific audiences through A/B testing [19].

With the RMTS approach, we move beyond A/B testing’s focus on “which advertisement pulls best” to a continuum of viewing experience that begins with a view or simple exposure as a baseline (obtaining attention), then progresses on to consider the percentage of a video watched—5%, 25%, 75%, or 90% (sustaining attention to conveying a message), and counts clicks on a call-to-action link (inspiring action) as a more meaningful way to measure user engagement. There is flexibility in this model to apply weighting to the viewing experiences of different audiences. For example, questions can be raised about whether younger viewers watch more or less of a video compared to older viewers, or whether younger people are more likely to click on the call-to-action link than older people.

In addition to conceptualizing age as a binary, we can also apply the same viewing experiences to gender or look at both gender and the gender-by-age interaction to gauge viewer experiences. With this data we can segment the audience based on their viewing experiences, a novel approach that not only delivers the optimized message to the audience most receptive to it but also considers viewing platforms or viewing style based on the user’s preference. Such an approach allows the content creator to choose the optimal combination of specific format attributes and formal features that can help the message resonate best among specific target audiences.

In our test of the RMTS, we were interested in exploring the impact of (1) the video’s aspect ratio (ie, rectangular, square, portrait), (2) the video’s length (short or long), and (3) the use of subtitles (present or absent) on the continuum of the viewing experience. Specifically, we raised the following research questions (RQs):

- RQ1: What is the impact of changing the aspect ratio of a video—a format attribute—on watch time and clicks on the call-to-action link?
- RQ2: How do formal features such as video length and subtitles impact watch time and clicks?
- RQ3: How do watch time and clicks of optimized videos compare to those of videos that have not been optimized?

We were also interested in examining the combination of video format attributes and formal features that led to higher abandonment rates for each of the 4 demographic segments in our study.

In summary, the purpose of this study was two-fold: (1) to demonstrate the use of the RMTS approach for message testing, especially when time and resources are limited such as during an infodemic, and (2) to propose and test the efficacy of an outcome variable that measures engagement along a continuum of viewing experience.

## Methods

### Study Design

This study is part of a larger vaccine confidence project we conducted in Odisha, India [20]. As of 2024, India is the country with the highest number of Facebook users in the world, with over 1 in 5 people using the platform [21]. As of February 2024, the largest Facebook user group in India were people aged 25-44 years, followed by people aged 18-24 years, while the majority of users (68%) identified as male [22].

### Intervention

For this study, we created 48 paid video advertisements on Facebook. The videos were all variations of one of the videos that we had produced for the larger vaccine confidence project to promote uptake of the COVID-19 vaccine, specifically the second dose. The original video length was 1 minute and 55 seconds and used narrative storytelling to address vaccine hesitancy. The story was set during a child’s birthday celebration. Mini, the protagonist, arrives late to the party and shares that she needs to leave early to go get the second dose of the COVID-19 vaccine. The video shows the conversation that ensues among family members. Mini explains the benefits of being vaccinated and counters the arguments made by Somnath, who plays the role of the vaccine skeptic, using a collective health frame. The video promotes the importance of being fully vaccinated against COVID-19 and includes a link that the viewer can click on to learn more and schedule an appointment to be vaccinated.

For this study, we created a shorter 58-second version of the video. For both the short and long versions of the video, we created a version that included subtitles in Odia, the language spoken in the video. By subtitles, we refer to captions or the accompanying text in the same language spoken in the videos that are shown near the bottom of the frame, imposed over a black gradient background to make them easier to read. The subtitles present the dialogue in text so that users who have their audio turned off, which is a popular behavior for users consuming content on their phones [14], can also follow the dialogue. The use of subtitles in our study is akin to the practice of close captioning, which has the additional benefit of making the content more accessible for Odia audiences who have difficulty processing audio content.

### Variables

The independent variables we looked at in our study were aspect ratio, subtitles, and length of videos. We took each of the 4 video variations (long or short, with or without subtitles) and configured them in 3 different aspect ratios: 16:9 (rectangle), 1:1 (square), and 4:5 (portrait) [23]. Table 1 shows the 12 video variations we tested in this study. We manipulated the length of the video, the aspect ratio, and the use (or lack of) subtitles. The original video was 1 minute 55 seconds long. The short version was 58 seconds long. We used the following Facebook specifications for testing the aspect ratio variable: rectangle (16:9), square (1:1), and portrait (4:5). All video advertisements included a call-to-action link that viewers could click on to find more information about vaccination.

**Figure 1:**
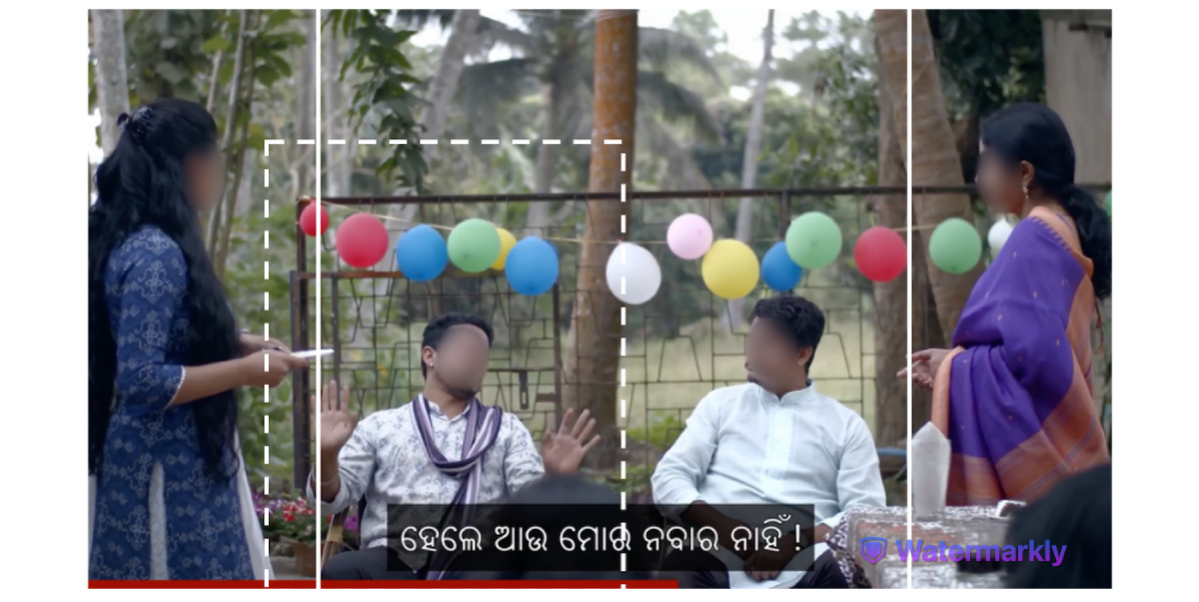
Impact of aspect ratio on image area visible to viewers.

Figure 1 shows how different aspect ratios affect the extent of the original image that is visible to the viewer: rectangular (the entire image), square (the portion of the image shown between the solid white lines), and portrait (the portion of the image shown within the dashed lines). The original video was shot in landscape orientation. The still captures the moment when Somnath (male, left), who played the role of the vaccine skeptic, gestures that he does not intend to receive the vaccine. The images include subtitles in Odia, the language spoken in the video [24]. In the portrait aspect ratio version of the video, the subtitles appear centered at the bottom of the screen.

In terms of demographic variables, the 12 variations we created for this study were tested with different audience demographics within the Odisha, India region. We used 4 age or gender combinations (ie, women or men, younger or older) equaling 48 unique advertisements. In our study, “younger” refers to users aged 18-22 years, and “older” refers to users aged 30-35 years, both falling within our age demographic of interest. We chose these 2 variables because age and gender are two of the most basic (and identifiable) demographic elements; these are the go-to variables as public health agencies would want to know how younger and older people, as well as men and women, differ. Each Facebook user within our target demographic was exposed to only 1 version of the video.

**Table 1:** Video variations by aspect ratio, presence or absence of subtitles, and video length.

Video variation number	Aspect ratio	Subtitles	Video length
1	Rectangle	No	Original
2	Rectangle	No	Short
3	Rectangle	Yes	Original
4	Rectangle	Yes	Short
5	Square	No	Original
6	Square	No	Short
7	Square	Yes	Original
8	Square	Yes	Short
9	Portrait	No	Original
10	Portrait	No	Short
11	Portrait	Yes	Original
12	Portrait	Yes	Short

### Measures

In our study, we were interested in measuring engagement along a continuum of viewing experience as our main outcome variable. Engagement was assessed as the number of seconds that the viewer watched the video and whether or not they clicked on the call-to-action link. We compared the results based on a continuum of viewing experience (ie, obtaining attention, sustaining attention, conveying a message, and inspiring action) to determine which approach would likely be more successful based on the criteria established for the test. We operationalized each step along the continuum of viewing experience as follows: obtaining attention (user watched 5% of the total video length), sustaining attention (user watched 25%-75% of the total video length), conveying a message (user watched 90% of the total video length), and inspiring action (user clicked on the call-to-action link). We compared the level of engagement garnered by the short video to the level of engagement garnered by the longer video. We were interested in learning how long viewers watched the video (watch time) and whether they clicked on the call-to-action link. We calculated the abandonment rate (AR) as follows:



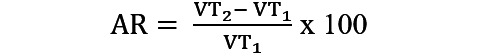



where VT_1_ represents the number of views at the time stamp closer to the start of the video and VT_2_ represents the number of views at the time stamp further from the start of the video. We were also interested in whether viewers clicked on the call-to-action link shown in the video as a measure of behavior related to the viewing experience.

### Statistical Analysis

For the statistical analysis, we ran a 3 (aspect ratio: rectangular, square, or portrait) × 2 (length: original or shortened) × 2 (presence or absence of subtitles) between-subjects experiment across 2 (age: younger or older) × 2 (sex: male or female) groups (for a total of 48 groups), using Meta’s A/B testing platform [25]. We used *χ*^2^ tests of independence to see if there were significant differences in the values observed across the variables of interest. In terms of sample size, N represents the total number of views and clicks for the 12 video variations, while n is used for the specific number of users watching the videos at 5%, 25%, 75%, and 90% watch time and the specific number of users who clicked on the call-to-action link. The results were considered significant when the probability or *P* value was .05 or less. Effect sizes were calculated using Cramér *V*, which is recommended for measuring the strength of association between nominal variables when the variables have more than 2 categories (the contingency table is larger than 2 × 2). Effect sizes were interpreted using Cohen’s [26] conventions for interpreting equivalent φ values. All statistics were calculated in Excel.

### Ethical Considerations

This study was approved by both the Institutional Review Board (IRB) at Johns Hopkins University in the United States (#18543) and the Sigma IRB in India (#10075). All data used in this study were collected through Meta’s A/B platform, which does not provide individual-level data. Therefore, the data are anonymized and aggregated upon receipt, without any personally identifiable information, and consistent with Meta’s data privacy and use policies.

## Results

**Figure 2:**
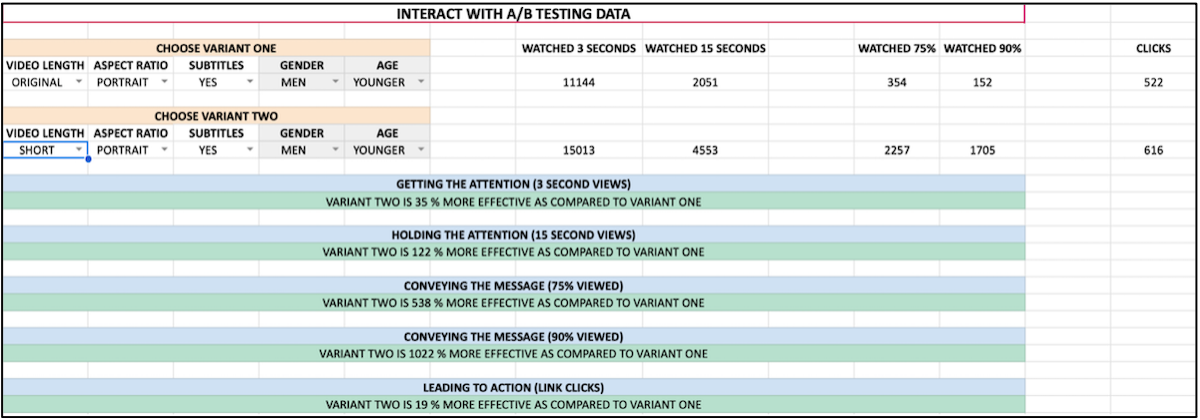
Screenshot of web-based dashboard comparing video variations.

The test was conducted over four consecutive days beginning in early August 2022 and cost US $450. The 48 video advertisements placed on Facebook garnered a total of 3.34 million impressions. The videos received a total of 700,239 views lasting at least 3 seconds (a 21% conversion rate from impressions); 182,890 views lasting at least 15 seconds; and a total of 25,270 clicks on the call-to-action link (3.6% of all 3-second views). The majority of viewers at 3 seconds of video play were men (399,703/700,239, 57%) and users aged between 30 and 35 years (444,164/700,239, 63%). Multimedia Appendix 2 contains the aggregate data from our study. We created a prototype for a web-based dashboard that would allow content creators to make evidence-based decisions based on the demographic segment (Figure 2). We have included a link to the web-based dashboard in the references section [27].

Table 2 shows the results of the *χ*^2^ tests by variable. In terms of RQ1, we observed that the video advertisements shown in the rectangle aspect ratio consistently experienced the highest abandonment rate, while the opposite was true for video advertisements shown in the portrait aspect ratio. In other words, the portrait aspect ratio seemed to retain viewers longer than other aspect ratios. The same was true for the numbers of clicks on the call-to-action link, with videos in the portrait aspect ratio yielding the greatest number of clicks, and videos in the rectangular aspect ratio yielding the least number of clicks.

In terms of RQ2, we observed that the short video, including both with and without subtitles, consistently outperformed the original (long) video across all audience segments. In fact, there was a strong and significant negative correlation between watch time and number of users watching, *r*(8)=–0.82, *P*=.01. In other words, as video play length increased, the number of users watching decreased. The abandonment rate for the original (long) video, both with and without subtitles, was almost 99% from 5% to 90% watch time, compared to 88% for the short video.

In terms of RQ3, the short video *without* subtitles attracted and retained more views longer than the short version *with* subtitles. We found that what we initially thought would be the optimal version (shorter, with subtitles and using the portrait aspect ratio) was not the best-performing version for all audience segments. We also found that, overall, the long video version with subtitles retained more views than the long version without them after the 25% watch time drop-off (n=15,597; χ21=7.33; *P*=.007; V=.02). This was true for all audience segments except younger women, for whom the short video version shown in portrait orientation and without subtitles performed better across the board.

**Table 2:** χ2 test results by variable. All results were significant (*P*<.001).

Variable		Views by percentage of video watched, n					Clicks, n		Chi-square (*df*)			Cramér *V*	
		5%	25%	75%	90%								
**Gender**										3999.68 (4)			0.06
	Women	300,536	89,090	37,162	26,136	11,831							
	Men	399,703	93,800	36,228	24,963	13,439							
**Age range (years)**										833.17 (4)			0.03
	18-22	256,075	68,710	26,370	18,227	11,358							
	30-35	444,164	114,180	47,020	32,872	13,912							
**Subtitles**										27.33 (4)			0.01
	Yes	347,714	89,763	36,402	25,129	12,328							
	No	352,525	93,127	36,988	25,970	12,942							
**Video length**										48,261.97 (4)			0.22
	Original	306,126	58,211	11,093	4504	11,736							
	Short	394,113	124,679	62,297	46,595	13,534							
**Aspect ratio**										3019.08 (8)			0.04
	Rectangle	227,017	54,638	19,610	13,387	7097							
	Square	233,754	59,997	23,967	16,524	8687							
	Portrait	239,468	68,255	29,813	21,188	9486							

## Discussion

### Principal Findings

Our main goal in this study was to introduce and apply a new approach for rapid message testing at scale that could be used by public health professionals to test and optimize content for public dissemination quickly and effectively during a public health crisis. The RMTS approach allowed us to test 48 social media advertisements on Facebook quickly and at a large scale. Overall, our results indicate that, regardless of aspect ratio manipulations and the presence or absence of subtitles, shorter videos, in portrait aspect ratio and without subtitles performed better across the board than other versions of the video. Our results are not surprising given what we know about how users tend to access social media on their mobile devices. The preference for portrait orientation could be because most users access Facebook on their smartphones, and most smartphone users hold their phones upright [28]. In that orientation, portrait aspect videos fill the screen and may create a more pleasant viewing experience without the user needing to expend additional energy tilting the device horizontally to view in landscape mode.

At the same time, it is not surprising that the shorter video version was able to retain more views than the original (longer) video version. Given what we know about distracted viewing and split attention among internet users, we do not interpret measures of viewing attention that are less than 100% as necessarily negative. While video producers and message sponsors strive to achieve 100% video completion rates, increased video abandonment rates have been a pesky issue for social media marketers. However, a video that is viewed 25% of the way through may not be unsuccessful, as the viewer may have gotten out of the video all that they wanted; the viewer perhaps clicked on the “learn more” button before the end of the video. However, it is also possible within this construct that a viewer decides to move on to other content after a few seconds of viewing.

In terms of subtitles, we were surprised to find that the video variations without subtitles performed better than those with subtitles. What we initially thought would be the optimal version (shorter, with subtitles and using the portrait aspect ratio) was not the best-performing version for all audience segments. Instead, the short video version shown in portrait orientation and without subtitles received the highest number of clicks across all audience segments. This is contrary to what we expected to find given industry reports citing the trend of users watching media on their mobile devices with the sound turned off. We thought the addition of subtitles would be beneficial to those users, but videos with subtitles had a higher abandonment rate at 25% watch time than those without. However, subtitles seemed to have helped retain more viewers and sustain their attention past the 25% watch time mark, except for younger women. Further research focusing on qualitative methodologies might shed light on these findings.

Our data suggests that on Facebook, shorter videos (<1 minute) shown in portrait orientation and without subtitles can lead to greater viewer engagement, but that subtitles may be beneficial in retaining views on longer videos (>1 minute). These findings may inform the construction of videos for dissemination on social media—editing the video for a shorter or perhaps longer beginning if, for example, the goal is to keep attention for as long as possible. At the end of the continuum is the behavioral outcome or action, which would take the form of a click on a “learn more” button embedded either in a video or video description. Further, while the ultimate measure may be whether the viewer clicks on the call-to-action link or not, we would not want to discount the other measures of attention offered by this continuum of viewing experience model based on viewer demographics of age and gender.

### Strengths and Limitations

The proposed RMTS approach satisfies some of the limitations present in A/B testing about the length of the test and the size of the sample. The issue regarding how long one should run an A/B test depends on the time and resources of the organization. Meta recommends that users of their A/B testing platform run their A/B tests for at least 2 weeks to 30 days [29]. However, depending on how many variations of an advertisement one is testing, this length of time can quickly add up. Furthermore, in a public health emergency, when time is of the essence, spending 2 weeks to a month just on testing is impractical. For all these reasons and working with a budget that we believed would be affordable (under US $500), we selected a 4-day test. During this 4-day interval, we garnered over 3 million impressions and over 1.3 million views beyond the first 3 seconds of the advertisements.

Our research study has several limitations. First, our research was limited to one social media platform, Facebook, and to the metrics provided by their A/B testing platform. We used Facebook because of the extent of its use among our target audience in India and because Meta’s A/B testing platform allowed us to test different variations of 1 video conveniently and cost-effectively. Our study looks at the demographic variables of age (young and old) and gender (male and female) only and does not integrate more diverse demographic variables that could offer a more nuanced understanding of viewer behavior. Unfortunately, these are the only demographic variables that we have access to through the Facebook A/B testing platform as part of their user privacy protections. We are also mindful of the fact that each platform has its own algorithmic bias and that the viewing experience is different depending on the platform. Prior research has shown the benefit of adopting a multi-platform approach that incorporates data from at least 2 social media platforms [30]. Given this limitation, we caution readers from extrapolating our Facebook findings to other social media platforms and recommend that future research incorporates a multi-platform approach. At the same time, the formats that we studied can be applied to other social media platforms such as Instagram, YouTube, and TikTok. We believe this approach can help organizations quickly and efficiently adapt messages across platforms without having to change the content to better target different audience segments, especially in time-sensitive situations when messages need to be disseminated quickly.

Another limitation is that when we shorten videos for testing as we did in this study, the longer and shorter versions vary on numerous dimensions and content, and hence a direct comparison might be difficult. This speaks to internal validity, as it affects our ability to make causal inferences. On the other hand, content creators also look for recommendations regarding the ideal length of video. Hence, if the recommendation is to make shorter videos, the underlying premise, of course, is that we have sacrificed content that would have been available in the longer format; that is the price we have to pay in comparing short and long videos.

Finally, while we found some significant associations between the length of the video, the use of subtitles, and the number of users watching the video at key points along the continuum of viewing experience, the effect size for those were overall very small. This means that our results have limited practical applications when it comes to the variables we tested. Maybe researchers looking at other forms of content beyond video advertisements on Facebook, and content variables other than aspect ratio and the use of subtitles, may find different and perhaps larger effect sizes. However, when it comes to population health interventions, Matthay et al [31] have noted that “even studies of interventions with small effect sizes can offer valuable evidence to inform population health if such interventions can be implemented broadly.”

### Future Research

Based on our study’s findings, we have several recommendations for future research. Given the reality of distracted viewing and attention spans on social media platforms that we mentioned, future research could assess the sustained impact of video advertisements on viewer behavior over time beyond initial engagement metrics. Our study focused on only 1 message to address vaccine hesitancy, with 1 protagonist (female), and using a collectivist appeal. We experimented with video length, aspect ratio, and subtitles. Future research could look at the impact of additional content variables such as different messages to address vaccine hesitancy, tone of voice, other forms of appeal, the identity of the protagonist, and cues such as music, to name some, that could also significantly impact viewer engagement. Our study was also limited to 1 social media platform, Facebook, which has its own unique algorithmic bias. At the most basic level, Meta’s A/B testing platform results we had access to were limited to binary gender data, excluding the perspectives of those who do not conform to those binary norms. Future research should look at incorporating other forms of content (beyond video advertisements) and applying the RMTS approach using other social media platforms.

This study is also limited by its quantitative methodology and its cultural specificity. To understand users’ more nuanced experience, we recommend that future research include a qualitative component in the methodology that explores how users from different backgrounds and identities consume videos, not just those whom Facebook identifies as young or old, female or male. Furthermore, our study was limited to Facebook users in Odisha, India. The content of the video itself was developed in collaboration with a Community Advisory Board in Odisha, and so, in a way, could be considered culturally specific. Future research should explore the impact that cultural differences have on message reception and engagement, especially given the global nature of social media platforms [32].

Finally, using social media for research can raise ethical considerations that researchers should carefully navigate, ranging from informed consent, privacy, data security, transparency, and accountability. For this study, we mentioned one of the limitations related to our limited demographic data. That, in itself, is a function of the process we used to collect the data. We worked exclusively through Meta’s A/B testing platform, which provides only anonymized and aggregated quantitative data in a way that conforms to Meta’s data policy. In other words, we have no way of identifying individuals based on the data we received. However, researchers looking at conducting studies with a strong qualitative component (eg, using comments and personally-identifying data posted on social media that is not meant to be shared outside that group or platform) should comply with their institutions’ IRB approval process, prioritize seeking informed consent from users, and afford the appropriate protections to participants following best practices for conducting qualitative research using social media [33-35]. In addition, special care needs to be taken when conducting tests during an infodemic, where there is already an overwhelming amount of information, some of which may be mis- or disinformation, competing for users’ attention and which could take away from the efforts of public health agencies. We believe this has to be balanced with conducting research whose findings can be critical in how subsequent crises are handled and for which communication guidelines are prepared. We hope that professionals working in public health will be able to use this RMTS approach to effectively respond and reach different publics during an unfolding public health crisis.

## Appendix

Multimedia Appendix 1 Glossary of terms related to the continuum of viewing experience.

Multimedia Appendix 2 Number of users who watched each video variation by age and gender, percentage of total video length played, and number of clicks (data file).

## Abbreviations

IRB: Institutional Review Board
RMTS: rapid message testing at scale
RQ: research question
UNICEF: United Nations Children’s Fund

## Data Availability

The data used in this study is included in Multimedia Appendix 2 as a .docx file. Please contact the corresponding author if you would prefer a .csv file.
