# Supplementary material for: Assessing Message Deployment During Public Health Emergencies Through Social Media: Empirical Test of Optimizing Content for Effective Dissemination
Source: J Med Internet Res. 2024 Jul 26;26:e50871. doi: 10.2196/50871 (PMC11316149; doi:10.2196/50871)
Supplement: Multimedia Appendix 1 [file jmir_v26i1e50871_app1.docx]

**Multimedia Appendix 1.** Glossary of terms related to the continuum of viewing experience.

Term Definition

**aspect ratio** the dimensions of an ad, expressed as width:height. For Facebook, the most common aspect ratios are 16:9 (rectangle or landscape), 1:1 (square), and 4:5 (portrait or vertical).

**abandonment rate (AR)** the drop in number of views as a video progresses from start to finish. This is similar to a drop-off rate, or people who start watching the video and stop watching before it is finished.

**click-through rate (CTR)** how often people click on a link in an ad to access additional content. It is calculated by dividing the total number of clicks for a post by the total number of impressions and multiplying that by 100.

**watch time** how long the user watched the video ad. In our study, it is expressed as a percentage of the total video length (i.e., 5, 25, 75, or 90%).

**vanity metrics** common metrics used to measure user engagement on social media platforms such as follows, likes, favorites, and emoji reactions. The term was coined by Rogers (2018).

**video length** the length of the video. In this study, we tested two video lengths: 58 seconds (short) and 1 minute, 55 seconds (long)

**video views** how many times a video has been played. On Facebook, a video must be played for at least 3 seconds to count as one view.
