# Supplementary material for: Assessing Message Deployment During Public Health Emergencies Through Social Media: Empirical Test of Optimizing Content for Effective Dissemination
Source: J Med Internet Res. 2024 Jul 26;26:e50871. doi: 10.2196/50871 (PMC11316149; doi:10.2196/50871)
Supplement: Multimedia Appendix 2 [file jmir_v26i1e50871_app2.docx]

**Multimedia Appendix 2.** Number of users who watched each video variation by age and gender, percentage of total video length played, and number of clicks (data file).

| Aspect  Ratio | Sub-titles | Video  Length | Gender | Age  Range | 5% | 25% | 75% | 90% | Clicks |
| --- | --- | --- | --- | --- | --- | --- | --- | --- | --- |
| Rectangle | No | Original | Women | 18-22 | 7815 | 2024 | 367 | 135 | 385 |
|  |  |  | Women | 30-35 | 15053 | 3089 | 580 | 258 | 453 |
|  |  |  | Men | 18-22 | 10344 | 1872 | 261 | 106 | 415 |
|  |  |  | Men | 30-35 | 18860 | 2884 | 417 | 213 | 476 |
| Rectangle | No | Short | Women | 18-22 | 10600 | 3535 | 1638 | 1255 | 420 |
|  |  |  | Women | 30-35 | 16751 | 5327 | 2563 | 1936 | 493 |
|  |  |  | Men | 18-22 | 12198 | 3124 | 1302 | 936 | 418 |
|  |  |  | Men | 30-35 | 23874 | 6272 | 2831 | 2062 | 519 |
| Rectangle | Yes | Original | Women | 18-22 | 8206 | 2152 | 423 | 138 | 339 |
|  |  |  | Women | 30-35 | 14962 | 3009 | 614 | 236 | 460 |
|  |  |  | Men | 18-22 | 9826 | 1730 | 226 | 93 | 371 |
|  |  |  | Men | 30-35 | 18732 | 2917 | 460 | 200 | 474 |
| Rectangle | Yes | Short | Women | 18-22 | 9324 | 2925 | 1443 | 1104 | 398 |
|  |  |  | Women | 30-35 | 15341 | 4722 | 2312 | 1757 | 467 |
|  |  |  | Men | 18-22 | 12188 | 3177 | 1348 | 962 | 475 |
|  |  |  | Men | 30-35 | 22943 | 5879 | 2825 | 1996 | 534 |
| Square | No | Original | Women | 18-22 | 7809 | 1862 | 368 | 142 | 448 |
|  |  |  | Women | 30-35 | 14034 | 2787 | 614 | 264 | 555 |
|  |  |  | Men | 18-22 | 9918 | 1713 | 228 | 105 | 464 |
|  |  |  | Men | 30-35 | 18579 | 2834 | 440 | 216 | 612 |
| Square | No | Short | Women | 18-22 | 10499 | 3844 | 1916 | 1479 | 494 |
|  |  |  | Women | 30-35 | 17982 | 6752 | 3544 | 2734 | 621 |
|  |  |  | Men | 18-22 | 13902 | 3882 | 1733 | 1232 | 556 |
|  |  |  | Men | 30-35 | 25911 | 7648 | 3585 | 2543 | 737 |
| Square | Yes | Original | Women | 18-22 | 7704 | 1829 | 429 | 142 | 393 |
|  |  |  | Women | 30-35 | 14061 | 2965 | 727 | 272 | 541 |
|  |  |  | Men | 18-22 | 9734 | 1646 | 280 | 96 | 444 |
|  |  |  | Men | 30-35 | 17802 | 2940 | 520 | 225 | 608 |
| Square | Yes | Short | Women | 18-22 | 11701 | 3867 | 1842 | 1387 | 488 |
|  |  |  | Women | 30-35 | 16612 | 5528 | 2926 | 2249 | 538 |
|  |  |  | Men | 18-22 | 13817 | 3442 | 1620 | 1151 | 535 |
|  |  |  | Men | 30-35 | 23689 | 6458 | 3195 | 2287 | 653 |
| Portrait | No | Original | Women | 18-22 | 8036 | 1913 | 420 | 163 | 474 |
|  |  |  | Women | 30-35 | 13558 | 2759 | 637 | 244 | 533 |
|  |  |  | Men | 18-22 | 10145 | 1727 | 281 | 109 | 527 |
|  |  |  | Men | 30-35 | 17765 | 2813 | 494 | 226 | 664 |
| Portrait | No | Short | Women | 18-22 | 11581 | 4633 | 2478 | 1904 | 582 |
|  |  |  | Women | 30-35 | 17412 | 6922 | 3755 | 2928 | 645 |
|  |  |  | Men | 18-22 | 14568 | 4552 | 2243 | 1676 | 644 |
|  |  |  | Men | 30-35 | 25331 | 8359 | 4293 | 3104 | 807 |
| Portrait | Yes | Original | Women | 18-22 | 8927 | 2314 | 543 | 204 | 451 |
|  |  |  | Women | 30-35 | 14687 | 3350 | 858 | 341 | 540 |
|  |  |  | Men | 18-22 | 11144 | 2051 | 354 | 152 | 522 |
|  |  |  | Men | 30-35 | 18425 | 3031 | 552 | 224 | 587 |
| Portrait | Yes | Short | Women | 18-22 | 11076 | 4343 | 2352 | 1851 | 499 |
|  |  |  | Women | 30-35 | 16805 | 6639 | 3813 | 3013 | 614 |
|  |  |  | Men | 18-22 | 15013 | 4553 | 2275 | 1705 | 616 |
|  |  |  | Men | 30-35 | 24995 | 8296 | 4465 | 3344 | 781 |
